# Current News and Opinion

**Published:** 1884-08

**Authors:** 


### Current News and Opinion.

### A FINE CHANCE.

The *Allgemeine Wiener Medicinische Zeitung*, contains the following advertisement in prominent type, with notice that applicants may obtain further information from the editor:

"A young unmarried doctor of medicine, of the Jewish persuasion, has the opportunity, by marriage and the succession to a lucrative dental practice, of insuring a very agreeable existence. The practice can be proved to be worth ten thousand florins per annum. The young lady in question is the daughter of highly respectable parents, seventeen years of age, handsome, well brought up, and equally well educated. Her father is desirous of retiring from the business, and will make over to a young doctor of medicine, without compensation, his dental practice and his excellent dwelling house, with all the material, dental instruments and apparatus, of the value of from eight to ten thousand florins. A knowledge of the dental art is not at first requisite, as under the tutelage of the present possessor this can be acquired in a few months."

This should be read in conjunction with the advertisement of a Canadian community, in which one church denomination was dominant, and in whose local paper a loud call was made for a "Baptist dentist." In the latter case, a "knowledge of the dental art" was probably not as essential as a firm belief in the doctrine of full immersion.

### PRE-HISTORIC DECAY OF THE TEETH.

The ancient Etrurians, who flourished in Italy before the founding of the Roman Empire, were in some respects the most remarkable people that ever existed upon the earth. Modern and recent excavations in their ancient burial places at Volterra, have resulted in the securing of great numbers of the teeth of this early race.

They give evidence of the most extensive decay, and of the existence of most of the modern dental diseases. Yet many writers and speakers who derive their facts from their theories, are continually referring to caries as the result of modern errors in diet, and a departure from the original state of nature in which man was made to live.

### THE SARATOGA MEETING.

In reply to the application of the Chairman of the Executive and Local Committees of the Association for reduced fares to Saratoga, the "Joint Executive Committee" of the various Passenger Lines has returned the following reply:

It will therefore be necessary for some member at each point referred to, to apply to the General Passenger Agent at his point of departure for more explicit information.

Persons attending the meeting from New York, via N. Y. C. & H. R. R. R. can purchase tickets for the round trip at $7.50.

The lines running east of New York, not being included in this combination—application must be made to their General Passenger Agents for rates.

We beg to call the attention of intending excursionists via Washington, Cincinnati, etc., to the advertisement of the New York, West Shore & Buffalo Railway Co. Passage by that route would doubtless be an improvement over any other.

MEYER L. RHEIN, Chairman Local Com.

FRANK M. ODFLL, Chairman Exec. Com.

OFFICE OF THE CHAIRMAN JOINT EX. COM.,
346 BROADWAY, NEW YORK, June 23d, 1884.

DR. M. L. RHEIN,
No. 7 West 38th Street, City.

DEAR SIR:

Referring to your application of the 2d inst., to Mr. E. J. Richards, A. G. P. A., for special fares to the meeting of the American Dental Association, Saratoga, August 4th to 9th, this committee in session last week decided that from the line of the following named roads:

| | |
|---|---|
| N. Y. C. & H. R. R. R. | N. Y., W. S. & B. R. R. |
| N. Y., L. E. & W. R. R. | D., L. & W. R. R. |
| Penn. R. R. | B. & O. R. R. |

covering the territory east of Buffalo, Salamanca, Pittsburg, Wheeling and Parkersburg, round trip tickets will be sold at the regular fares for summer tourist tickets. From the territory west of the points above named to Chicago, Cincinnati and St. Louis delegates will be furnished round trip tickets at one and one third the lowest unlimited fares; such tickets to be sold August 1st to 5th inclusive, good for return passage to starting point to and including August 15th, 1884.

I would refer you to the General Passenger Agents of the lines interested in the business for any further information necessary to carry out the details connected with the transportation of your people to the meeting.

Yours truly,
R. T. REYDER, Secretary.

(14.) Sometimes a pulp partly devitalized, or a fragment of pulp, will remain in so sensitive a condition as to defy efforts to remove or fully destroy it. Will some one please inform me what to do in such cases ? NEW JERSEY.

---

(15.) We sometimes find the permanent incisors in mouths of young people rough or serrated on their cutting edges. Is it best to grind them smooth, or leave them unmolested ? G. B.

---

(16.) I have used Listerine and find it a pleasant preparation; but I would like to be informed if it is *really* an "antiseptic" or "germicide." T——.

---

(17.) Dentists talk about "*perfect* fillings," and "perfectly *solid* gold fillings." I wonder if such fillings are ever made? C. F. M.

---

(18.) Cannot somebody give us a little treatise on fixtures for retaining teeth in their new positions after regulating? The process is nearly useless without this, and yet the books almost ignore it. D.

---

(19.) In the penetration of tooth structure does the germ precede the acid, or vice-versa ? This was one of the interesting questions at the last meeting of the Mass. Den. Soc.—but no satisfactory conclusion was arrived at.

I enclose you an anomaly in the shape of a third bicuspid which occupied the place of the second permanent molar (left superior). All of the other teeth were regular and perfect. This one had been exceedingly sore and troublesome for a long time, and the patient (a physician) would consent to no treatment other than extraction. C. W. STARBUCK.

---

*Answers.*—Reply to F. S. (No. 11).

FORMULA FOR GOLD SOLDER.

| | |
|---|---|
| 18 K. gold plate scraps, | 9 pwts. |
| Brass wire, . . | 3 " |
| Silver, . . . | 1 " |

Roll thin. This will flow smoothly and evenly if the piece is well heated before the blow-pipe is used. It will leave so good a finish that little dressing down will be needed. R. N. LAWRANCE, Lincoln, Ill.

---

In response to M. W. L. (No. 13), permit one of your readers to state, that careful observation during a practice of thirty years has led to the conclusion that tobacco neither benefits nor injures the teeth. It, however, gives the breath a decidedly unpleasant taint, and when used immoderately seems to permeate the entire human structure. W. D.

